# Water Quality, Heavy Metals, and Antifungal Susceptibility to Fluconazole of Yeasts from Water Systems

**DOI:** 10.3390/ijerph20043428

**Published:** 2023-02-15

**Authors:** Luz Dary Caicedo-Bejarano, Lizeth Stefania Osorio-Vanegas, Mauricio Ramírez-Castrillón, Jorge Enrique Castillo, Carlos Andrés Martínez-Garay, Mónica Chávez-Vivas

**Affiliations:** 1 Research Group in Mycology (GIM/CICBA), Facultad de Ciencias Básicas, Universidad Santiago de Cali, Santiago de Cali 760035, Colombia; 2 Department of Biochemical Engineering, Faculty of Engineering and Design, Universidad Icesi, Santiago de Cali 760031, Colombia; 3 Grupo de Investigación en Electroquímica y Ambiente (GIEMA), Facultad de Ciencias Básicas, Universidad Santiago de Cali, Santiago de Cali 760035, Colombia; 4 Grupo de Investigación GIMMEIN, Programa de Medicina, Facultad de Salud, Universidad Libre Seccional Cali, Santiago de Cali 760031, Colombia

**Keywords:** wastewater, drinking water, yeasts, susceptibility, antifungals, water quality, heavy metals

## Abstract

Aquatic environments could be reservoirs of pathogenic yeasts with acquired antifungal resistance. The susceptibility to antifungal agents of yeasts present in the wastewater and natural waters of the city of Cali was evaluated. Samples were taken from two types of water: drinking water (Meléndez River, drinking water treatment plant “Puerto Mallarino” in the Cauca River) and wastewater (South Channel of the Cauca River, “Cañaveralejo-PTAR” wastewater treatment plant). Physico-chemical parameters, heavy metal concentration, and yeast levels were determined using standard procedures. Yeasts were identified using API 20 C AUX (BioMérieux) and sequence analysis of the ITS1-5.8S-ITS2 and D1/D2 regions of the large subunit of the ribosome. Susceptibility assays against fluconazole and amphotericin B using the minimum inhibitory concentration (MIC) test were determined using the microdilution method. The influence of physico-chemical parameters and heavy metals was established using principal component analysis (PCA). Yeast counts were higher at WWTP “PTAR” and lower at Melendez River, as expected. A total of 14 genera and 21 yeast species was identified, and the genus *Candida* was present at all locations. Susceptibility tests showed a 32.7% resistance profile to fluconazole in the order DWTP “Puerto Mallarino = WWTP “PTAR” > South Channel “Navarro”. There were significant differences (*p* < 0.05) in the physico-chemical parameters/concentration of heavy metals and yeast levels between the aquatic systems under study. A positive association was observed between yeast levels and total dissolved solids, nitrate levels, and Cr at the “PTAR” WWTP; conductivity, Zn, and Cu in the South Channel; and the presence of Pb in the “Puerto Mallarino” DWTP. *Rhodotorula mucilaginosa*, *Candida albicans*, and *Candida* sp. 1 were influenced by Cr and Cd, and *Diutina catelunata* was influenced by Fe (*p* < 0.05). The water systems explored in this study showed different yeast levels and susceptibility profiles, and, therefore, possible genetic differences among populations of the same species, and different physico-chemical and heavy metals concentrations, which were probably modulating the antifungal-resistant yeasts. All these aquatic systems discharge their content into the Cauca River. We highlight the importance to further investigate if these resistant communities continue to other locations in the second largest river of Colombia and to determine the risk posed to humans and animals.

## 1. Introduction

Yeasts constitute an important component in the microbial community of aquatic environments and are comparable to other microorganisms such as bacteria and protozoa [1,2]. Aquatic environments and sediments are important habitats to study microorganism diversity, and yeasts can be easily found in these ecosystems, presenting phenotypic plasticity and adaptations to tolerate salinity, environmental temperature, oxygen saturation, and acidity [3,4,5]. In this sense, several pathogenic or opportunistic yeasts can be isolated from lakes [6,7,8,9], rivers [7,9,10], drinking water [7,11], and wastewater [1,4,5,12]. They are susceptible to acquiring resistance to medical antifungals, such as fluconazole, which is the most widely used agent in the clinic as primary treatment [13], and triazole fungicides, which are frequently used in agriculture [14], while some of them have intrinsic resistance, such as the species *Candida krusei*, *Rhodotorula mucilaginosa*, and *Meyerozyma guilliermondii* [15,16]. Furthermore, yeasts can acquire secondary resistance due to previous contact with antifungals in patients, or low drug activity in tissue or blood associated with immunosuppressed patients [17,18]. The increase in fungal infections has been accentuated by antifungal drug resistance being a serious public health problem. Reports of yeast strains resistant to commonly used antifungal drugs in aquatic environments are becoming more frequent, so these environments can present potential health risks to people who use contaminated water. For example, Medeiros et al. [9] found isolates of *Candida* spp. resistant to itraconazole and fluconazole in the lakes and rivers of southeastern Brazil. Milanezi et al. [4] conducted a study in the Rio Grande do Sul, Brazil, and found a high frequency of *Candida* spp. and *Debaryomyces* spp. resistant to azole antifungals and amphotericin B. Several studies reported the isolation of pathogenic yeasts resistant to antifungal agents from lakes, lagoons, rivers, and wastewater treatment plants (WWTP), mainly in South America [6,8] and South Africa [1,7,10,12]. In Colombia, the intensive use of azole antifungals without medical prescription, or their use as a prophylactic in immunosuppressed patients, increased the number of cases of resistant yeasts, mainly of *C. albicans* [19] or the *C. parapsilosis* complex [20]. The approach to this problem has focused mainly on studies from the clinical point of view [19,20,21]. Even though a high load of antifungals in the environment has been documented, more than 80% are azoles. This can be explained to the wide use in clinical therapy and the use of fungicides due to its low cost and systemic action, which allows both the prevention and treatment of fungal diseases, long-lasting stability in the environment and broad antifungal spectrum. [14,17,20,21,22]. Several studies around the world have revealed high levels of antifungal azoles in the aqueous phases of WWTP effluents, after their use in homes, hospitals, or agricultural industries [22,23], and a low elimination rate of these antifungals has been reported [24,25]. At the same time, some yeasts isolated from contaminated water have the potential in biotechnological processes to produce ethanol, biodiesel, and carotenoid pigments, among others [26]. Therefore, the objective of this study was to determine yeast levels in aquatic environments in the city of Cali, Colombia, and their fluconazole and amphotericin b susceptibilities. Furthermore, we aimed to establish their associations with physico-chemical parameters and heavy metal concentrations, with the purpose of driving further research and public policy interventions to control the quality of aquatic systems and strengthen the response to fungal infections and antifungal resistance.

## 2. Materials and Methods

### 2.1. Study Area and Sampling

**Figure 1:**
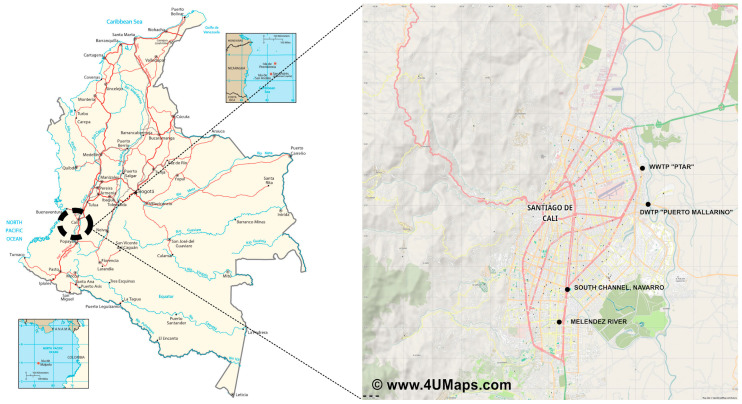
Location of the sampling stations. South Channel: rainwater canal; DWTP: drinking water treatment plant “Puerto Mallarino”, Cauca River; WWTP: wastewater treatment plant “PTAR”; and Melendez River. The map on the right was modified from www.4UMaps.com (accessed on 21 October 2022) under the license Data CC BY-SA by OpenStreetMap.

The study was performed in Cali (at coordinates 3°27′00″ N 76°32′00″ W), a city located in southwestern Colombia. The weather of the city is warm, and in the course of the year, the temperature generally varies from 19 °C to 30 °C. Water samples were collected from four water systems: Melendez River (south of the city), the intake from the drinking water treatment plant (DWTP) “Puerto Mallarino” (intake is located on the Cauca River and supplies 76% of drinking water to the city), and wastewater samples were taken from a rainwater channel in Navarro, called “South Channel” (this channel also receives wastewater from a part of the community in the south of the city), and the municipal WWTP (best known as “PTAR”), which discharges its water into the Cauca River, the main river of the city and the second biggest river in Colombia. A schematic illustration of the sampling sites and how they are connected is shown in Figure 1. For fungal isolation, the water samples were taken in triplicate using a plastic container tied to a rigid support, in a sterile 500 mL amber glass container, protected with an aluminum seal and/or Teflon cap, fully filled in January 2018 (dry season) and May 2018 (wet season). The samples were taken at a depth of approximately 0.5 m and as far as possible from the shore, trying not to disturb the bottom and avoiding backwaters or stagnant areas, and were immediately sent to the laboratory in a cooler to be processed and to start the analysis within six hours of taking the sample. One liter (in triplicate) of collected water samples was divided into two equal portions of 500 mL, one of which was stabilized with nitric acid for the determination of heavy metals, while the other was used for the analysis of physico-chemical parameters; samples were kept at 4 °C for no more than two days before being analyzed [27].

### 2.2. Yeast Isolation and API20C Tests

Serial dilutions (10^−1^–10^−3^) were made with peptone water (Oxoid Ltd., Hampshire, United Kingdom). Equal volumes (200 μL) from each dilution were spread over the surface of Dicloran Rose Bengal Chloramphenicol (DRBC) (Merck, Darmstadt, Germany) and CHRomagar Candida™ agar plates (BBL International Inc., London, UK). All the plates were incubated for 48 h at 30 °C. Colony counts were performed on individual plates. Representative yeast colonies were selected and grouped by morphotype, isolated, and conserved using Saboraud Dextrose Agar (Merck) immersed in sterile mineral oil and cryopreserved in glycerol 30% (*v*/*v*). A macroscopic evaluation of colonies was performed with a stereoscope (Celestron Lab’s S10-60 Stereo, Celestron, LLC, Torrance, CA, USA) taking account of the shape, edge, surface, appearance, elevation, brightness, and consistency. All the yeasts were evaluated for sugar assimilation using the API 20C AUX system (BioMérieux, Marcy l’Etoile, France) according to the manufacturer’s instructions. Several carbohydrates were evaluated, including D-Glucose, Glycerol, 2-keto-Gluconate calcium, L-Arabinose, D-Xylose, Adonitol, Xylitol, D-Galactose, Inositol, D-Sorbitol, Methyl-D-Glucopyranoside, N-Acetyl-Glucosamine, D-Lactose (Bovine), D-Maltose, D-Sucrose, D-Trehalose, D-Melezitose and D-Raffinose [28]. The galleries were incubated at 30 °C for 72 h in an airtight box containing a small volume of water to create a humid atmosphere. The observation of yeast growth was considered positive. The negative control contained no carbon source, and the positive control contained glucose. The carbohydrate assimilation profile obtained for each tested isolate was interpreted using ApiwebTM software (BioMérieux, reference: 40011).

### 2.3. Molecular Identification

For DNA extraction, each yeast liquid culture was centrifuged for 2 min at 12,000× *g* and the supernatant was discarded. DNA was extracted using Kit GeneJet (Thermo Fisher, Waltham, MA, USA) and resuspended in 50 μL of TE (10 mM Tris, 1 mM EDTA, pH 7.4). DNA was visualized on a 1% (*w*/*v*) agarose gel and its concentration and purity were determined using a Nanodrop 2000 spectrophotometer (Thermo Scientific, Waltham, MA, USA). The ITS1-5.8-S-ITS2 (Internal transcribed spacer, ITS) and D1/D2 domain of the large subunit of the ribosome (LSU) regions were amplified using the polymerase chain reaction (PCR) procedure in a final volume of 25 μL. A total of 5 μL of genomic DNA (approximately 1 ng/μL) was taken and resuspended in 20 μL of the PCR mixture comprising 0.4 μL primer Forward 20 pmol/μL, 0.4 μL primer Reverse 20 pmol/uL, 1 μL 1 mM dNTPs, 1.5 mL MgCl2 50 mM 0.2 μL sterile milli-Q H2O, 0.6 μL dimethyl sulfoxide (DMSO), 0.2 μL of Taq Polymerase (5 U/μL) (Bioline, London, United Kingdom), and 2.5 μL of 10X Buffer. We used ITS5/ITS4 primers for the ITS region and NL1/NL4 primers for the LSU region. PCR products were analyzed using 1.5% (*w*/*v*) agarose gel electrophoresis in 1X TAE buffer (tris base, acetic acid, EDTA, distilled water), and run at 100 V for 1 h. To visualize band migration, the gel was stained with SybrGreen (LONZA) and observed under UV light. A 100-bp or 1-kb ladder (Gibco BRL, Burlington, ON, Canada) was used to estimate amplicon size. Amplification products were purified and sequenced on an ABI 3130 Genetic Analyzer (Applied Biosystems, Waltham, MA, USA) at CorpoGen company. Subsequently, the sequences were edited, assembled, and compared in Genbank and Mycobank databases using the Basic Local Alignment Search Tool (BLAST) algorithm. A 98.41% or 99.5% threshold was used for identification at the taxonomic level of species for ITS and LSU, respectively [29].

### 2.4. Antifungal Susceptibility Tests to Fluconazole and Amphotericin B

The antifungal susceptibility test was performed using the broth microdilution technique, following the M27-A3 guidelines from the Clinical and Laboratory Standards Institute (CLSI) [30]. Amphotericin B (AMPB, Sigma Chemical Co., St. Louis, MO, USA) was dissolved in dimethyl sulfoxide and fluconazole (FCZ, Pfizer Central Research) was suspended in sterile water. Microtiter plates with 96 round-bottom wells were prepared with a range of final concentrations from 0.03 to 16 μg/mL for Amphotericin B and from 0.125 to 64 μg/mL for FCZ. Serial two-fold dilutions of the various drugs were prepared in RPMI 1640 medium (with L-glutamine, without bicarbonate; Sigma Chemical Co.) and buffered to pH 7.0 using 0.165 M [N-morpholino]propane-sulfonic acid solution (MOPS; Sigma Chemical Co.). The yeast suspension was made at a McFarland concentration of 0.5 (adjusted to final concentrations of approx. 2.5 × 10^3^ CFU/mL). The inoculated plates were incubated for 24–48 h at 35 °C. Strains *C. krusei* ATCC 6258 and *C. parapsilosis* ATCC 22,019 were used as controls to detect any abnormalities and deactivation of the antifungal. To determine the MIC, the concentration that produced a reduction in yeast growth of ≥50% compared to control growth without FCZ and total absence of growth for AMPB after 48 h of incubation was considered. Interpretations of the results were based on breakpoints provided by M57S and M27M44S of CLSI [30,31]. Isolates were classified as sensitive (S), dose-dependent sensitive (DDS), and resistant (R).

### 2.5. Analysis of Physico-Chemical Parameters and Heavy Metals

Physico-chemical parameter analyses (temperature, pH, turbidity, and electrical conductivity) were determined in situ using a 350 multi-parameter probe (Merck, Germany). Water samples were transported on ice to the laboratory to determine salinity, solid particles, dissolved oxygen, total phosphorus, and nitrites according to the standard methods described in the current National Drinking Water Quality Standard (GB5749). The concentrations of the seven heavy metals iron (Fe), copper (Cu), lead (Pb), cadmium (Cd), zinc (Zn), silver (Ag), and chromium (Cr) were determined using a flame atomic absorption spectrophotometer (Model ZEEnit 700P, Analytik Jena, Germany).

### 2.6. Statistical Analysis

Correlation tests were performed to investigate the relationship between physicochemical parameters, heavy metal concentrations, and fluconazole susceptibility values for the different yeast species. Principal component analysis (PCA) was performed to determine patterns and relationships in the data. Data output was provided in the form of correlation biplots using the packages FactoMineR and ggrepel for R v4.1.1. The correlation significance level was set at *p* < 0.05. A comparison between the different sampling points for each variable was performed using Kolmogorov–Smirnov tests (*p* < 0.05).

## 3. Results

**Figure 2:**
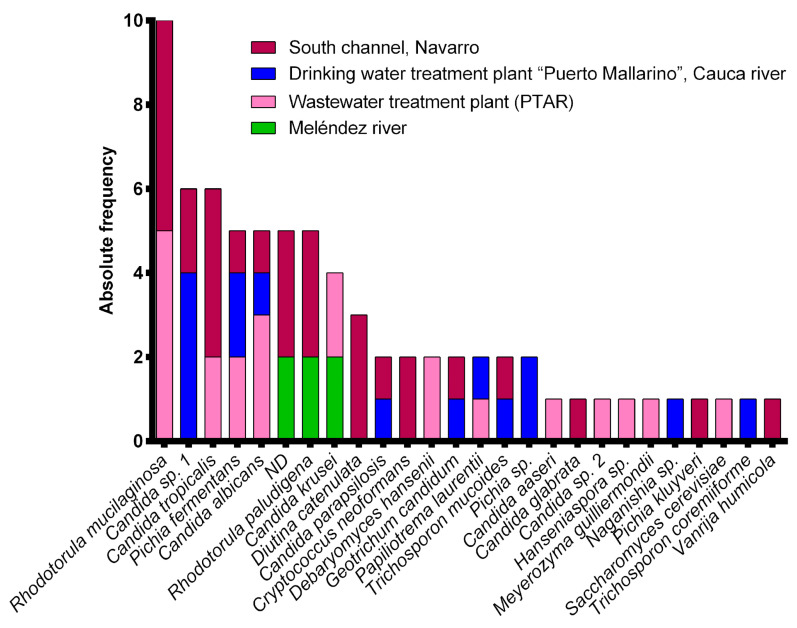
Absolute frequency of yeast species found in different aquatic systems in Cali, Colombia. ND = not determined.

Yeast counts were different for each sample when cultured on a DRBC medium. There were no significant differences in the number of yeasts at each sampling site during the dry and wet seasons. A higher concentration of yeasts was identified in the intake wastewater from the “PTAR” WWTP (2.4 × 10^5^ CFU/mL), while the Melendez River had a lower concentration (<1.0 × 10^3^ CFU/mL). The “Puerto Mallarino” DWTP and South Channel presented intermediate concentrations of yeasts (7.7 × 10^4^ CFU/mL and 3.6 × 10^4^ CFU/mL, respectively). In the present study, we recorded a total of 73 yeast isolates from all sources. Sixty-eight yeast isolates were identified, belonging to 14 genera and 21 species; five isolates were not identified (Table S1). The genus *Candida* represented most of the yeast isolates. It was interesting that elevated levels of species belonging to *Candida* (8 × 10^3^ CFU/mL) were also determined in the samples from the “PTAR” WWTP. Chromagar Candida medium showed colonies of three morphotypes: *C. albicans*, *C. tropicalis*, and *Pichia kudriavzevii* (syn. *C. krusei*). Among the yeast species identified, *Rhodotorula mucilaginosa* (13.7%), *Candida* sp. 1 (8.2%), and *C. tropicalis* (8.2%) were the most frequent (Table S1, Figure 2). However, several isolates were not identifiable to the taxonomic level of species according to the criterium established by Vu et al. [29] using sequence analysis. In this sense, six strains marked as *Candida* sp. 1 were closely related to *C. intermedia*, while the strain *Candida* sp. P46 (*Candida* sp. 2) was closely related to *C. pseudolambica*. The strain *Naganishia* sp. PM20 was related to *N. diffluens* and two strains of the genus *Pichia* were related to *P. fermentans*, with 94.87% of sequence identity compared to the type strain of the species.

The antifungal susceptibility was assessed in 49 yeast isolates after 24–48 h of growth. The reading of the appearance of the biomass was carried out with the help of an inverted mirror. The minimum inhibitory concentration (MIC) breakpoints for FCZ for *C. albicans*, *C. parapsilosis*, and *C. tropicalis* isolates with MIC ≤ 2 µg/mL were considered susceptible, those with MIC 4 µg/mL were considered susceptible dose-dependent (DDS), and those with MIC ≥ 8 µg/mL were considered resistant; *C. glabrata* isolates with MIC ≤ 32 µg/mL were considered DDS, while isolates with MIC ≥ 64 µg/mL were considered resistant. All *P. kudriavzevii (C. krusei)* and *Rh. mucilaginosa* isolates were considered resistant regardless of the MIC value. In the case of amphotericin B, yeasts with an epidemiological cutoff value MIC ≤ 2 µg/mL were considered susceptible.

Yeast isolates with sensitivity to FCZ corresponded to 49%, followed by resistant isolates (32.7%), and dose-dependent sensitive isolates (18.4%). All yeast strains were sensitive to AMPB (Table 1). Inside each aquatic system, the ratios of susceptibilities were different for FCZ. In the South Channel, the ratio of sensitivity was higher (59.1%) compared to other susceptibilities, with the least resistance to FCZ (27.3%). On the other hand, the ratio of sensitivity from the “Puerto Mallarino” DWTP was 36.4% and the percentage of resistant yeasts was 36.4%. This resistant ratio was similar at the “PTAR” WWTP (35.7%). We found variations in sensitivity to FCZ among *C. tropicalis* isolates according to the site sampled; for example, at South Channel, there were sensitive and DDS strains, but at “PTAR” WWTP we found sensitive and resistant strains (i.e., P4). A similar trend was observed for *C. albicans*. For example, strains CS55 (South Channel) and P1 (WWTP) showed an MIC of 64 and 8 μg/mL, respectively. However, strains P13B (WWTP) and PM22 (DWTP) were DDS and sensitive, respectively, suggesting variability among populations of this species.

**Figure 3:**
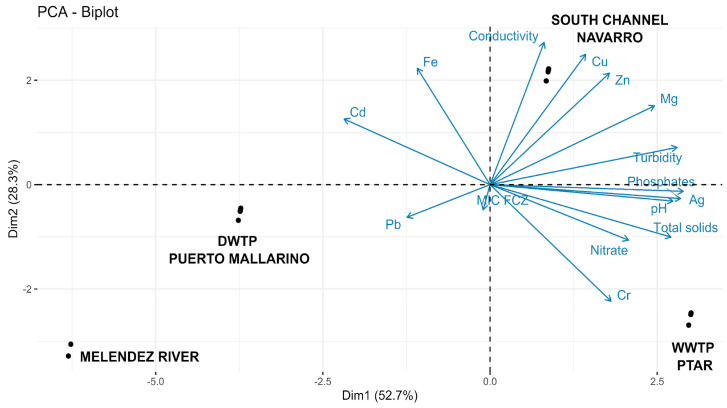
Principal component analysis correlation biplots of the interaction of physico-chemical parameters, heavy metal concentrations, yeast levels, and the association of the various sampling points (C = rainwater canal “South Channel”; PM = DWTP “Puerto Mallarino”; P = WWTP “PTAR”).

In the present study, PCA was used to establish associations between physico-chemical parameters, the concentrations of heavy metals, and yeast levels from different sampling sites. Table 2 shows the correlation between heavy metals and physico-chemical parameters of water samples and the MIC of FCZ-resistant yeasts. High correlation values (R^2^ > 0.5, *p* < 0.05) were obtained for the ratio of iron to *T. mucoides* and *D. catenulate*; zinc to *C. parapsilosis* and *D. catenulata*; iron to *C. parapsilosis*; Cd to *C. albicans*, *Rh. mucilaginosa*, and *Candida* sp 1; phosphates to *D. hansenii*; nitrates to *Rh. mucilaginosa*; pH to *C. tropicalis* and *D. hansenii*; conductivity to *C. parapsilosis*; and temperature to *C. parapsilosis* and *C. albicans*. Figure 3 depicts the distribution of the physico-chemical parameters and heavy metal concentration variables formed by the first two axes, which explained 81.0% of the total variance. A positive association was observed between yeast levels with total dissolved solids and nitrate levels in the WWTP, and with conductivity in the South Channel. The correlation between the levels of antifungal-resistant yeasts and the presence of heavy metals varied between the sampling sites. Yeast levels of *C. albicans*, *C. parapsilosis*, *Candida* sp. 1, *P. fermentans*, *T. coremiiforme*, *P. laurentii*, and *G. candidum* were influenced by the presence of Pb in the “Puerto Mallarino” DWTP. Furthermore, *C. albicans*, *C. tropicalis*, *C. krusei*, *C. aaseri*, *Candida* sp. 2, *P. fermentans*, *S. cerevisiae*, *P. laurentii*, and *D. hansenii* were influenced by Cr in the “PTAR” WWTP. Finally, *C. parapsilosis*, *C. tropicalis*, *C. krusei*, *C. albicans*, *Candida* sp. 1, *P. fermentans*, *D. catenulata*, *Rh. mucilaginosa*, *Cr. neoformans*, *V. humicola*, and *G. candidum* were influenced by Zn and Cu in the South Channel. We also found significant correlations between specific heavy metals and some specific fluconazole-resistant yeast species.

## 4. Discussion

Aquatic environments can become polluted because of various anthropogenic activities, such as agricultural, industrial, and medical waste production, the expansion of urban communities without adequate sanitation infrastructure, and poor wastewater management [1,10,12]. One of the main indicators of water quality is microorganisms, the main concern being water intended for human consumption and recreational activities, without neglecting surface water, groundwater, and wastewater [32]. Some studies have indicated that the increased contamination of aquatic environments was significantly positively correlated with the relative abundance of yeasts; however, few studies have been conducted to assess the quality of aquatic ecosystems contaminated with yeasts [33,34]. In this study, yeasts were detected at all sampling sites, including the intake of the “Puerto Mallarino” DWTP. At this plant, the order of the process carried out to treat the water is as follows: collection, sand removal, application of activated carbon, pre-chlorination, coagulation, flocculation, sedimentation, and filtration. In addition, there is post-chlorination and chemical stabilization with lime. However, the use of chlorine alone does not guarantee the absence of pathogenic fungi in drinking or recreational water. In fact, Ma et al. showed that there were non-significant changes in fungal community structure observed before and after the initiation of treatment of a hospital hot water system treated with monochloramine in situ [35].

This DWTP follows the parameters established in Colombia, according to decree 1575 of 2007 [36] and resolution 2115 of 2007 [37], that determine the physico-chemical and microbiological quality control of drinking water. The physico-chemical analysis includes the parameters of pH, color, turbidity, nitrate, fluoride, and residual chlorine, among others. Microbiological standards include the absence of pathogenic microorganisms and fecal bacteria (*Escherichia coli* and *Enterococcus* spp.), and the determination of the presence of *Giardia* sp. and *Cryptosporidium* spp. It should be noted that fungi are not included as potential water contaminants in these documents. The possible reasons for this may be the lack of knowledge of the fungal load in the water, the use of divergent culture methods, the heterogeneous mechanisms of pathogenicity of the fungi, and, consequently, the low number of reports connecting the presence of fungi in the tap water and the appearance of diseases in humans as stated by Kauffmann-Lacroix et al. [38]. Our results suggest that regulations about microbiological procedures should be updated and yeast counting included in routine analyses.

The number of yeasts was significantly higher in the “PTAR” WWTP than Melendez River. The variability of microbial counts found in the present study could be explained by the different levels of water quality of these environments, as they receive different kinds and levels of pollutants in the form of wastewater, industrial effluents, and others. However, the number of heterotrophic yeasts in the Melendez River (1.0 × 10^3^ CFU/mL) was higher than that reported in other rivers around the world, such as Rio Doce, Brazil (from 10 to 466 CFU/mL) [9], some rivers in South Africa (Mooi River (from 0.5 to 9 CFU/mL) [11] and Eersterivier River catchment (1.0 × 10^2^ CFU/mL)) [39], the River Danube in the area of Bratislava (100–210 CFU/mL) [40], and a lake in Patagonia, Argentina (22–141 CFU/mL) [41]. However, at the Tagus estuary, Portugal, a higher level of yeasts was reported (5.3–3272 CFU/mL) [42]. We highlight that the threshold of environmental quality in the eutrophic ecosystem was above 1.0 CFU/mL) [33].

An important aspect was the isolation of *C. albicans*, *C. krusei*, and *C. parapsilosis*, which are related to a high burden of fungal infections in the healthcare environment in Colombia [21]. The highest number of the genus *Candida* (8 × 10^3^ CFU/mL) was counted at the “PTAR” WWTP. Our results were consistent with the findings of Assress et al. [12], who also found that *Candida* spp. were the principal species in WWTPs in Gauteng, South Africa. The results demonstrated the attractiveness of using yeasts as a microbiological indicator of organic pollution in aquatic ecosystems [12]. Regarding pigmented yeasts, we found only yeasts from the genus *Rhodotorula*, with high carotenoid production in almost all isolates [26]. *Rh. mucilaginosa* was found only at the “PTAR” WWTP and the South Channel in high numbers. These yeasts are associated with skin diseases, and mucosal and invasive fungal infections, especially in immunosuppressed patients, and they present a special metabolic activity in highly eutrophicated waters or severely contaminated municipal wastewaters, which is indicative of their association with anthropogenic activity [43]. Studies on the evaluation of the risk of infection by different species of environmental yeasts has been little described in Colombia. For example, *Rhodotorula* spp. have been isolated from the nails of patients with superficial mycosis in Antioquia, Colombia [44]. *Cr. neoformans* is mainly associated with the droppings of various bird species, especially pigeons, but is also isolated from bark, tree trunk hollows, and decaying wood [45], and has been reported in wastewater in previous studies [46]. *Cr. neoformans* causes opportunistic infections that usually affect the central nervous system of generally immunosuppressed patients, with high mortality in patients who do not receive treatment [47]. In Colombia, the main challenge to treat infections caused by this yeast is resistance to fluconazole [48]. *M. guilliermondii* is a globally distributed opportunistic pathogen that lives in various habitats. It exists on human skin and in the surface microbiota of mucous membranes. It can cause serious fungal infections such as candidemia [49]. It is a yeast considered a promising species in the field of biotechnology, especially in the biocontrol of moldy bacteria during the storage of fruits and vegetables [50]. The presence of these yeasts is understandable, since city wastewater effluents containing antimicrobials, antimicrobial resistance-carrying microbes, and antimicrobial resistance genes are discharged at these two sites. Among the most reported opportunistic yeasts we found *M. guilliermondii*, *C. lusitaniae*, *C. tropicalis*, *P. laurentii*, *Rh. glutinis*, *and Rh. mucilaginosa* in surface waters [11,39].

The main fungal pathogens in humans are *Candida* species, which develop resistance to triazoles and echinocandins. However, resistance to AMPB (Polyene) is extremely rare, despite its use for more than 60 years as a monotherapy, mainly for invasive fungal infections [51,52]. For this reason, it is interesting to know whether amphotericin B-resistant yeast strains can be found in the environment. The results obtained with the MIC assay showed that all strains of the different yeast species (including *Candida* species) were found to be sensitive to AMPB, with values at or below 2 μg/mL, confirming that resistance to this antifungal is rare in the assessed yeasts, and suggesting a low interaction of this antifungal with yeast communities in this location. This was not the case for FCZ, where 32.7% of yeast species were resistant to FCZ. These results were consistent with results obtained in previous studies focusing on the isolation of antifungal-resistant environmental yeast. Brilhante et al. reported azole resistance in *Candida* spp. isolated from Catú Lake, Ceará, Brazil in a high number [53]. Similarly, Medeiros et al. reported in 2008 that 50% of the yeasts isolated from water samples of the lakes and rivers of the Rio Doce, Brazil showed resistance to itraconazole and less resistance to FCZ [54]. However, an evaluation carried out in 2012 showed significant growth of yeast resistant to FCZ [9]. The yeasts isolated at the South Channel (Navarro) showed the highest percentage of resistance (37.5%) to FCZ, possibly associated with the high load of contaminants that this canal receives along the south of the city.

Table 1 illustrates the pathogenic species *C. tropicalis*, *C. albicans*, *C. parapsilosis*, *D. catelunata*, *Rh. mucilaginosa*, and *D. hansenii*, showing the highest MIC values to FCZ (64 μg/mL). Similarly, antifungal resistance to FCZ has been reported in *Candida* species from tropical freshwater environments in Brazil and China [9,53,54,55]. The presence of environmental yeasts with high resistance to FCZ is probably due to the fact that this azole is the most widely used antifungal in humans and animals, and it is among the most reported drugs in hospital and home wastewater [56,57]. In addition, there are fungicides routinely used in agriculture that share the action mechanism of azoles, which can generate cross-resistance in yeasts found in these aquatic environments [56,57,58]. In Asia, some studies reported a significant increase in resistant *C. tropicalis* isolated from poultry, and this has been associated with the extensive use of azoles in agriculture [56,59,60]. Brilhante et al. suggested that azole resistance in the *Candida* strains recovered from aquatic environments would be influenced by the activity of efflux pumps [53]. Several chemical compounds present in the environment, when interacting with microorganisms, would trigger the expression of genes that lead to these efflux pumps or other proteins involved in resistance to antifungals [56,57,58].

*Rhodotorula*, *Candida*, *Pichia*, and *Trichosporon* are yeasts typical of strongly eutrophic waters [61], and their presence indicates that aquatic systems are significantly polluted by industrial and municipal wastewater, where they are able to metabolize aromatic substances and heavy metals and have been considered as good indicators of pollution [1,8,33,62]. It is not surprising to find these yeasts in the environment; however, in a report by the WHO, which included a list of fungal priority pathogens, it recommended carrying out this type of study in order to provide epidemiological data that allow the establishment of local priorities in relation to the dynamics of fungal pathogens and the prevalence of resistance to reduce the impact of drug resistance worldwide [63].

The yeast species compositions in the aquatic systems studied were dissimilar and were conditioned by the type of water, but also by the processing conditions. Yeast culture is an effective way to study their diversity, as many more yeast species, especially some possible new species, were recorded and isolated using the culture method. We found an association between yeast levels with certain physico-chemical parameters, such as total dissolved solids, levels of nitrites, and conductivity in the “PTAR” WWTP and South Channel. These results can be explained by the high load of waste that these two places receive, especially due to domestic discharges and agricultural activities that cause an increase in the concentration of organic matter and salts that come from fertilizers. Bafico demonstrated in his study that pollution from urban sources strongly influences the periphytic community structure and dynamics of Lake Nahuel Huapi (Patagonia, Argentina), with greater biomass and cell densities at highly contaminated sites [41,64]. In addition, the highest number of fluconazole-resistant yeast isolates was detected at these two sites (31.3% and 37.5%, respectively). The observed spatial variation could be attributed to site-specific anthropogenic activities, such as eutrophication, the influx of domestic and industrial waste, and run-off from agricultural settings. Effluents from the Melendez River, rainwater at the South Channel (Navarro), and the WWTP (PTAR) discharge into the Cauca River. In addition, the vicinity of the river presents different levels of human interactions (e.g., illegal mining, steel making, informal settlements, sewage and raw pharmaceuticals discharge, and poultry, agricultural, and industrial waste), which influence yeast counts and the species present, and the activation or acquisition of antifungal resistance mechanisms.

The yeast count obtained during the dry and wet season did not present significant differences at each sampling site, with the reported number corresponding to the average of the two sampling moments. Colombia, being a tropical country, experiences changes in climatic conditions that are conditioned by two phenomena, the “La Niña” phenomenon (there can be rain all year with some sunny days) and the “El Niño” phenomenon (there can be dry days all year with some rainy days). This means that temperature and relative humidity conditions each year are not very variable. Precipitation in January and May 2018 was 50–100 mm and 100–150 mm, respectively, with average temperatures for the two months between 20 and 28 °C [65]. The yeast count obtained during the dry and wet season did not present significant differences at each sampling site, with the reported number corresponding to the average of the two sampling moments. For this reason, the data from the two sampling moments were averaged. The results differed from those found by Steffen et al., who found significant differences in site-specific yeast concentrations in the dry summer months, when concentrations increased significantly during the rainy months [39], and from the results of Ortíz-Vera et al., who found greater differences in community composition (relative abundance of fungal phyla) in the dry than in the rainy season [66].

On the other hand, heavy metal pollution in aquatic systems is a global problem originating from increased industrialization and urbanization, since they are accumulative, toxic, and carcinogenic in water bodies and biota. As seen from the result of the PCA, there was a significantly positive correlation between levels of *Candida* spp., *P. fermentans*, among others, and heavy metals, especially Cr, Zn, Cu, and Pb. There is little open literature on the effect of metal levels on the diversity and structure of the fungal community in aquatic environments. Assress et al. found that the distribution of classes such as *Pezizomycetes*, *Lecanoromycetes*, *Agaricostilbomycetes*, *Schizosaccharomycetes*, and *Dothideomycetes* was influenced by Mg and Zn concentrations [12]. Meanwhile, members of classes *Eurotiomycetes*, *Exobasidiomycetes*, *Orbiliomycetes*, *Glomeromycetes*, *Saccharomycetes* and *Leotiomycetes* were correlated with Ni and Mn levels, and Fe was the main environmental parameter influencing the fungal community belonging to classes *Agaricomycetes*, *Pucchinomycetes*, *Atractiellomycetes*, *Sordariomycetes*, and *Archaeorhizomycetes* in WWTPs located in Gauteng Province, South Africa.

Some studies have shown that the content of organic matter, calcium, and amorphous phase can increase the sensitivity to heavy metal contamination and the persistence of organic pollutants in different matrices (water, air, and soil), and interfere or inhibit some enzymes and metabolic processes [67].

It is also established that the organic matter content is known to positively influence the cation exchange capacity, buffering capacity, and retention of heavy metals; for example, some physico-chemical factors, such as pH and oxidation reduction potential, can influence the increased toxicity and genotoxicity of some yeasts to heavy metals [68,69].

It has been shown that the presence of heavy metals in aquatic environments influences the structure of bacterial communities resistant to antibiotics and the permanence over long periods of time of genes resistant to antibiotics by forming stable complexes of antibiotics and metal ions [70]. Dickinson et al. demonstrated the co-selection of heavy metals and antibiotics in the environment [71]. The prevalence and persistence of resistant genes to antibiotics and heavy metals have been demonstrated in WWTPs [72]. High levels of antibiotic resistant genes are reported to be related to high concentrations of Cr [73], Hg, and Zn [74]. Rajasekar et al. found that Cd, Pb, and Cu had a significant positive correlation with the *sul2* and *strB* genes, conferring resistance to sulfonamides and streptomycin, respectively [75]. This would explain the results of our study, where it was found that *Rh. mucilaginosa*, *C. albicans*, and *Candida* sp. 1 were positively associated with the presence of Cd, and *D. catelunata* was associated with Fe.

*Saccharomyces cerevisiae* and some pathogenic yeasts, such as *C. glabrata* and *C. albicans*, have been reported to have ATP-binding cassette (ABC) transporters, which have evolved to play critical roles in adapting to environmental challenges such as heavy metal stress and multidrug resistance (PDR). For example, Yap1, Yap2, and Yap8 transporters regulate the response to oxidative and heavy metal stress by inducing the expression of the yeast activator protein YCF1, the pleiotropic drug resistance elements PDR5, and the multidrug transporter involved in multidrug resistance and singlet oxygen species resistance (SNQ2) in response to a variety of toxic metals. Yeast ABC transporters play an important role in plasma membrane homeostasis, the site of action of antifungals, such as polyenes and azoles, and are involved in resistance to different drugs, which could explain the correlation between the presence of some heavy metals and the susceptibility to fluconazole of some yeasts isolated in this study [76,77]. In relation to the presence of heavy metals in soils, *Suillus luteus* uses different mechanisms, such as the exclusion and regulation of heavy metals through transporters located in the cell membrane and chelating agents, which are released to the outside of the cell wall, through storage, and the trapped ions are stored or transported in vesicles and expelled from the cells, in the same way that oxidizing agents relieve oxidative stress caused by reactive oxygen species through reducing agents such as cytochromes p450 (CYP450) [78]. CYP450 exists in a large number of organisms, including bacteria, fungi, and mammals, catalyzing a variety of oxidation reactions that allow the detoxification of xenobiotics, such as heavy metals, the metabolism of drugs, such as antifungals, and the biosynthesis of steroids, such as ergosterol [79]. One of these antifungals, fluconazole, acts on ergosterol biosynthesis in fungal cells by inhibiting a CYP450-dependent fungal enzyme, lanosterol 14α-sterol demethylase. Resistance to fluconazole can occur through the increased expression of the ERG11 gene encoding the drug target enzyme, sterol 14α-demethylase (Erg11p), or by mutations in *Erg11p* that result in a reduced affinity for fluconazole and an overexpression of efflux pumps that transport fluconazole out of the cell [80].

However, more extensive studies are needed to determine the influence of physico-chemical parameters, heavy metals, and other contaminating compounds in yeasts resistant to Cd antifungals in water systems.

## 5. Study Limitations

Our study was based on cultivated yeasts, which limited the diversity analysis, so it will be important in subsequent studies to carry out metagenomic shotgun and metatranscriptome experiments that would allow us to further explore the mycodiversity and functional genes that are being expressed in water systems.

It is also important to include other antifungals in the antimicrobial susceptibility analysis, and to perform an evaluation of the virulence profile, such as the production of hydrolytic enzymes, and the adhesion capacity and formation of biofilms, which would allow us to clarify the possible route of transmission of these pathogens to humans.

## 6. Conclusions

We found opportunistic yeast pathogens, such as strain-specific azole-resistant *C. albicans*, *C. parapsilosis*, *C. tropicalis*, and *Candida* sp. 1 (closely related to *C. intermedia*), in different aquatic environments, including a WWTP and DWTP. Melendez River was an exception. These results are disturbing since this drug is frequently used as a prophylactic treatment in HIV-positive patients. We emphasized the importance of carrying out studies focusing on the isolation of antifungal-resistant environmental yeasts to investigate their participation in the deterioration of aquatic environments.

The results of the correlation analysis showed a general spatial variation In the characteristics of the wastewater samples. Yeast counts and some species considered opportunistic pathogens, such as *C. albicans*, *Rh. mucilaginosa*, and *D. catelunata*, showed an association with parameters and chemical compounds indicative of the presence of sewage or contaminated water, which act as selection agents for pathogenic yeasts.

The results indicated that the aquatic systems of the city of Cali are a reservoir of FCZ-resistant yeasts and a potential source of invasive fungal infections, which is important for the “One Health” approach, and, like other ecosystem services, these must be protected and managed in a sustainable manner.

## Figures and Tables

**Table 1:** Minimal inhibitory concentrations (MIC) of yeasts associated with water systems in Cali against Fluconazole (FCZ) and Amphotericin B (AMPB).

Strain	Species	Susceptibility to Fluconazole	FCZ MIC (μg/mL)	AMPB MIC (μg/mL)
**CS11**	*Rh. paludigena*	Sensitive	2	2
**CS14**	ND	Sensitive	2	2
**CS15**	*V. humicola*	Sensitive	0.5	0.25
**CS16**	*C. parapsilosis*	Sensitive	2	0.5
**CS17**	*D. catenulata*	Sensitive	0.5	1
**CS18**	*D. catenulata*	Sensitive	0.5	0.5
**CS19**	*C. tropicalis*	DDS	4	2
**CS1A**	*C. glabrata*	DDS	4	0.5
**CS1B**	*Rh. mucilaginosa*	Resistant *	1	2
**CS21**	*P. fermentans*	DDS	4	2
**CS22**	ND	Sensitive	0.25	2
**CS23**	*Cr. neoformans*	Sensitive	0.125	2
**CS24A**	*Cr. neoformans*	Sensitive	2	1
**CS4**	*T. mucoides*	Sensitive	2	2
**CS45**	*P. kluyveri*	Sensitive	0.5	0.5
**CS51A**	*D. catenulata*	Resistant	8	2
**CS51B**	*C. tropicalis*	Resistant	16	2
**CS55**	*C. albicans*	Resistant	64	2
**CS7**	*C. tropicalis*	Sensitive	1	2
**CS7B**	*G. candidum*	Resistant	2	2
**CS7C**	ND	Resistant	64	2
**CS9**	*Candida* sp. 1	Sensitive	0.25	1
**M2**	ND	Sensitive	2	2
**M12**	ND	Resistant	64	2
**P1**	*C. albicans*	Resistant	8	1
**P13B**	*C. albicans*	DDS	4	1
**P14**	*C. aaseri*	Sensitive	0.5	0.5
**P16A**	*C. tropicalis*	Sensitive	2	2
**P16B**	*P. laurentii*	Sensitive	0.5	2
**P20A**	*D. hansenii*	DDS	4	2
**P22**	*P. kudriavzevii (C. krusei)*	Resistant *	4	1
**P24A**	*D. hansenii*	Resistant	64	0.5
**P24B**	*Rh. mucilaginosa*	Resistant	64	2
**P3**	*S. cerevisiae*	DDS	4	2
**P3A**	*Hanseniaspora pseudoguilliermondii*	Sensitive	1	2
**P4**	*C. tropicalis*	Resistant	64	0.5
**P46**	*Candida* sp. 2	Sensitive	0.25	2
**P9A**	*P. fermentans*	Sensitive	2	2
**PM14**	*G. candidum*	Sensitive	0.5	1
**PM15**	*Pichia* sp.	DDS	4	2
**PM18**	*T. coremiiforme*	Sensitive	2	0.5
**PM19**	*P. fermentans*	DDS	4	2
**PM20**	*Naganishia sp.*	Sensitive	0.125	2
**PM22**	*C. albicans*	Sensitive	0.5	2
**PM24**	*Candida* sp. 1	DDS	4	2
**PM4A**	*T. mucoides*	Resistant	8	2
**PM4B**	*P. laurentii*	Resistant	16	2
**PM54**	*Candida* sp. 1	Resistant	64	0.5
**PM54A**	*C. parapsilosis*	Resistant	16	2

* Due to intrinsic resistance, these strains were marked as resistant, despite the observed MIC. Yeast strains labeled with “CS” were isolated from the South Channel, Navarro; yeast strains labeled with “P” were isolated from the “PTAR” WWTP; yeast strains labeled with “PM” were isolated from the “Puerto Mallarino” DWTP, Cauca River; and yeast strains labeled with “M” were isolated from the Melendez River. ND: not determined. DDS: dose-dependent Sensitive.

**Table 2:** Correlations between physico-chemical parameters, metal concentrations, and MIC of fluconazole-resistant yeasts.

Heavy Metals/ Parameters	*Cp*	*Ct*	*Ca*	*Pk*	*Rhm*	T*m*	*Gc*	*Dh*	*Pl*	*Dc*	*C*sp*1*
Fe	**0.469**	0.090	0.251	0.192	−0.816	0.543	0.352	−0.135	0.406	**0.816**	0.333
Ag	0.020	0.404	0.249	0.095	0.000	−0.089	−0.027	**0.456**	−0.188	**−0.816**	0.333
Zn	0.506	0.269	**0.470**	0.095	**−0.816**	−0.134	0.027	0.026	0.295	**0.816**	−0.333
Cu	**0.506**	0.000	0.249	0.190	**−0.816**	−0.134	0.188	−0.134	0.188	**−0.816**	**−0.666**
Cd	**−0.292**	−0.324	**0.805**	0.024	**0.816**	0.208	0.000	−0.194	−0.388	**−0.816**	**0.912**
Cr	−0.179	0.173	0.000	0.079	0.500	−0.198	−0.208	**0.505**	−0.148	**−1.000**	−0.182
Pb	−0.066	0.172	0.331	0.239	0.000	0.172	0.147	0.000	−0.147	0.000	0.333
Mg	0.303	0.224	0.359	0.190	**−0.816**	−0.044	0.080	0.080	0.134	0.000	−0.333
TP	0.060	**0.449**	0.304	−0.047	0.000	−0.269	−0.241	**0.510**	−0.134	**−0.816**	0.000
Turbidity	0.263	0.224	0.359	0.191	**−0.816**	0.000	0.080	0.080	0.080	0.000	0.000
TDS	0.183	−0.022	**−0.700**	−0.024	**−0.816**	−0.135	−0.027	−0.054	0.135	0.000	**−0.666**
Nitrate	−0.060	−0.045	−0.415	−0.429	**0.816**	**−0.674**	−0.403	0.134	−0.188	**−0.816**	**−1.000**
NT	0.101	0.314	0.304	0.000	**−0.816**	0.000	−0.134	0.241	0.026	0.000	0.333
pH	0.067	**0.522**	0.464	0.237	0.000	−0.124	0.029	**0.534**	0.029	0.000	−0.235
COND	**0.604**	0.074	0.402	0.342	**−0.816**	0.322	0.386	−0.178	0.326	0.000	−0.235
TEMP	**0.469**	0.372	**0.526**	0.289	**−0.816**	0.024	0.207	0.178	0.148	0.000	−0.235

*Cp*: *Candida parapsilosis*,*Ct*: *C. tropicalis*, *Ca*: *C. albicans*, *Rhm*: *Rhodotorula mucilaginosa*, *Pk*: *Pichia kudriavzevii* (*C. krusei*), *Tm*: *Trichosporon mucoides*, *Gc*: *Geotrichum candidum*, *Dh*: *Debaryomyces hansenii*, *Pl*: *Papiliotrema laurentii*, *Dc*: *Diutina catenulata*, *Csp1*: *Candida* sp. 1. TP = total phosphorus, TDS = total dissolved solids, NH4 = nitrate, TN = total nitrogen, COND = conductivity, TEMP = temperature. The values in bold present a statistically significant (*p* < 0.05) positive or negative correlation between variables.

## Data Availability

Data sharing not applicable.

## Supplementary Materials

The following supporting information can be downloaded at: https://www.mdpi.com/article/10.3390/ijerph20043428/s1, Table S1: Yeast strains were analyzed in this study. All samples were identified with the biochemical tests API 20C and were confirmed using sequence analysis. Yeast strains labeled with “CS” were isolated from the South Channel, Navarro; yeast strains labeled with “P” were isolated from WWTP “PTAR”; yeast strains labeled with “PM” were isolated from DWTP “Puerto Mallarino”, Cauca River; and yeast strains labeled with “M” were isolated from Melendez River. ND: not determined. ITS = Internal Transcribed Spacers; LSU = D1/D2 domain of the Large Subunit of the Ribosome. References [26,81] are cited in the Supplementary Materials.

## Abbreviations

The following abbreviations are used in this manuscript:

WWTP	Wastewater Treatment Plant
DWTP	Drinking Water Treatment Plant
PTAR	Planta de Tratamiento de Aguas Residuales
ITS	Internal Transcribed Spacer 1, gene rDNA 5.8S and Internal Transcribed Spacer 2
LSU	D1/D2 Domain of the Large Subunit of the Ribosome 26S
CFU	Colony Forming Units
MIC	Minimal Inhibitory Concentration
FCZ	Fluconazole
AMPB	Amphotericin B
PCA	Principal Component Analysis
