# Supplementary material for: Water Quality, Heavy Metals, and Antifungal Susceptibility to Fluconazole of Yeasts from Water Systems
Source: Int J Environ Res Public Health. 2023 Feb 15;20(4):3428. doi: 10.3390/ijerph20043428 (PMC9968106; doi:10.3390/ijerph20043428)
Supplement: Supplementary file 1 [file ijerph-20-03428-s001.zip › ijerph-2156793-supplementary.pdf]

International Journal of
Environmental Research and Public Health

MDPI

Supplementary Material

Table S1. Yeast strains were analyzed in this study. We used two methods of identification: Biochemical tests (API 20C) and sequence analysis. Yeast strains labeled with "CS" were isolated from the South Channel, Navarro; yeast strains labeled with "P" were isolated from WWTP “PTAR”; yeast strains labeled with "PM" were isolated from DWTP “Puerto Mallarino”, Cauca River; and yeast strains labeled with "M" were isolated from Melendez River. ND: not determined. ITS= Internal Transcribed Spacers; LSU= D1/D2 domain of the Large Subunit of the Ribosome.

**Strain**	**Species**	Identification **method**	**GenBank Accession**	Reference
CS11	*Rhodotorula paludigena*	ITS	MT161369	[26]
CS12A	*Rh.mucilaginosa*	ITS	-	[26]
CS13	*Rh.paludigena*	ITS	MT161370	[26]
CS14	ND	-	-	
CS15	*Vanrija humicola*	API 20C	-	
CS16	*Candida parapsilosis*	API 20C	-	
CS17	*Diutina catenulata*	ITS	OP696682	
CS17A	*Rh.mucilaginosa*	LSU	OP658772	[26]
CS18	*D. catenulata*	ITS	OP696683	
CS19	*C. tropicalis*	LSU	OP658773	
CS1A	*C. glabrata*	API 20C	-	
CS1B	*Rh. mucilaginosa*	ITS	-	[26]
CS2	*Candida* sp. 1[a]	ITS	OP696681	
CS20A	*C. tropicalis*	LSU	OP658775	
CS20B	*Rhodotorula paludigena*	ITS	MT161371	[26]
CS21	*Pichia fermentans*	ITS	OP696684	
CS22	ND	-	-	
CS23	*Cryptococcus neoformans*	API 20C	-	
CS24	*Cr. neoformans*	API 20C	-	
CS4	*Trichosporon mucoides*	API 20C	-	
CS45	*P. kluyveri*	ITS	OP696685	
CS51A	*D. catenulata*	ITS	-	
CS51B	*C. tropicalis*	API 20C	-	
CS55	*C. albicans*	ITS	OP696686	
CS7	*C. tropicalis*	ITS/LSU	OP658776	
CS7B	*Geotrichum candidum*	API 20C	-	
CS7C	ND	-	-	
CS9	*Candida* sp. 1	ITS	MK256275	
CS9A	*Rh. mucilaginosa*	ITS	MT161372	[26]
CS9B	*Rh. mucilaginosa*	ITS	OP696687	[26]
M10	*Rh. paludigena*	ITS	-	
M12	ND	-	-	
M14	*P. kudriavzevii*	ITS/LSU	OP658777	
M2	ND	-	-	
M23A	*P. kudriavzevii*	LSU	OP658778	
M23B	*Rh. paludigena*	ITS	MT161375	[26]

P1	*C. albicans*	ITS	OP696688	
P10A	*Rh. mucilaginosa*	ITS	MT161376	[26]
P10B	*Rh. mucilaginosa*	ITS	-	[26]
P12	*P. kudriavzevii*	ITS/LSU	OP658782	
P13A	*Candida* sp. 1	ITS	MK256275	
P13B	*C. albicans*	API 20C	-	
P14	*C. aaseri*	ITS/LSU	OP658783	[81]
P15	*P. fermentans*	ITS	-	
P16A	*C. tropicalis*	API 20C	-	
P16B	*Papiliotrema laurentii*	ITS	-	
P20A	*Debaryomyces hansenii*	API 20C	-	
P22	*P. kudriavzevii*	API 20C	-	
P24A	*D. hansenii*	ITS	-	
P24B	*Rh. mucilaginosa*	ITS	OP696689	
P3	*Saccharomyces cerevisiae*	API 20C	-	
P3A	*Hanseniaspora pseudoguilliermondii*	LSU	OP658784	
P4	*C. tropicalis*	API 20C	-	
P46	*Candida* sp. 2[b]	ITS	MK256277	
P5	*Meyerozyma guilliermondii*	ITS	-	
P7	*Rh. mucilaginosa*	LSU	OP658843	
P8	*Rh. mucilaginosa*	ITS	-	
P9A	*P. fermentans*	ITS	OP696690	
PM14	*G. candidum*	API 20C	-	
PM15	*Pichia* sp.	ITS	OP696691	
PM18	*T. coreemiforme*	ITS	OP696692	
PM19	*P. fermentans*	ITS	OP696693	
PM20	*Naganishia* sp.	ITS	OP696697	
PM22	*C. albicans*	API 20C	-	
PM24	*Candida* sp. 1	ITS	-	
PM4A	*T. mucoides*	API 20C	-	
PM4B	*P. laurentii*	API 20C	-	
PM54	*Candida* sp. 1	ITS	MK256279	
PM54A	*C. parapsilosis*	API 20C	-	
PM59	*Candida* sp. 1	ITS	MK256278	
PM64	*Candida* sp. 1	ITS	MK256276	
PM79	*P. fermentans*	ITS	OP696694	
PM7B	*Pichia* sp.	ITS	OP696695	

[a] Closely related to *Candida intermedia*. [b] Closely related to *Candida pseudolambica*
